# Factors that influence contraceptive use amongst women in Vanga health district, Democratic Republic of Congo

**DOI:** 10.4102/phcfm.v6i1.599

**Published:** 2014-05-15

**Authors:** Kangale Izale, Indiran Govender, Jean-Pierre L. Fina, John Tumbo

**Affiliations:** 1 Department of Family Medicine, Protestant University of Congo, Democratic Republic of Congo; 2 Department of Family Medicine and Primary Health Care, University of the Limpopo, Medunsa Campus, South Africa

## Abstract

**Background:**

Contraception is often necessary in order to plan for children and without it there is a risk of unplanned pregnancies. In the Democratic Republic of Congo, this often results in abortions by untrained persons with resultant morbidity and mortality.

**Aim:**

To investigate the factors that influence contraceptive use amongst women of childbearing age in the Vanga health zone.

**Methods:**

Cross-sectional survey using interviewer-administered questionnaires.

**Results:**

Of the 384 women recruited, a majority (46.1%) were in the 31–40 year age group; 52% had reached primary school and 88% did not have formal employment. One hundred and forty of the participants reported current use of contraception, with 60% of them using modern methods of contraception; 36.1% of them had begun using contraception before the age of 20; and the most common methods were oral contraceptive pills and injection, each accounting for 22.9%. There was variation in the duration of contraceptive use and the main reason for using contraception was to space children. Of the participants, 20.7% had been using contraception for more than two years. Seventy-seven (31.5%) of the women reported they did not use contraception because of a fear of side effects. Forty-four (18%) reported that they are unable to afford contraception, 38 (15.6%) had husbands who disapproved of contraceptive usage, 26 (10.6%) had a fear of infertility, 18 (7.4%) practised a religion that did not allow them to use contraception and 12 of the women (4.9%) did not use contraception because it was unavailable to them.

**Conclusion:**

Barriers to contraception in our study were fears of side effects and infertility, cost, male partner's objection, unavailability of contraception and religious beliefs.

## Abstrait

**Les facteurs qui influencent l'utilisation des contraceptifs par les femmes dans le district de santé de Vanga, en République Démocratique du Congo**.

**Contexte:**

La contraception est souvent nécessaire pour la planification familiale, et sans elle on risque des grossesses non planifiées. En République Démocratique du Congo cela donne souvent lieu à des avortements par des personnes non qualifiées conduisant à la morbidité et mortalité.

**Objectif:**

Etudier les facteurs qui influencent l'utilisation des contraceptifs chez les femmes en âge d'avoir des enfants dans la zone de santé de Vanga.

**Méthodes:**

Etude transversale utilisant des questionnaires remplis par l'enquêteur.

**Résultats:**

Des 384 femmes recrutées, la majorité (46.1%) appartenait au groupe d’âge de 31 à 40 ans; 52% avaient été à l’école primaire et 88% n'avaient pas d'emploi dans le secteur structuré. Cent quarante des participantes ont déclaré qu'elles utilisaient des contraceptifs, dont 60% utilisaient des méthodes modernes de contraception; 36.1% avaient commencé à utiliser la contraception avant l’âge de 20 ans; les méthodes les plus courantes étaient les pilules contraceptives orales et les injections, comptant chacune pour 22.9%. Il y avait une différence dans la durée d'utilisation des contraceptifs et la raison principale pour leur utilisation qui était l'espacement des naissances. Parmi les participantes, 20.7% utilisaient la contraception depuis plus de deux ans. Soixante-dix-sept femmes (31.5%) ont déclaré qu'elles n'utilisaient pas la contraception par crainte des effets secondaires. Quarante quatre (18%) ont déclaré qu'elles n'avaient pas les moyens d'utiliser la contraception, 38 (15.6%) avaient des maris qui étaient contre la contraception, 26 (10.6%) avaient peur de l'infertilité, 18 (7.4%) pratiquaient une religion qui ne leur permettait pas d'utiliser la contraception et 12 femmes (4.9%) n'utilisaient pas la contraception parce qu'elle n’était pas disponible pour elles.

**Conclusion:**

Dans notre étude les barrières à la contraception étaient la crainte des effets secondaires et de l'infertilité, le coût, l'objection du partenaire mâle, la non disponibilité de la contraception et les croyances religieuses.

## Introduction

Women worldwide are exposed to the risk of unwanted and unintended pregnancies as a result of ineffective or non-use of contraception. Women who are able to use known contraceptive methods are able to decide if and when they want children, as well as how many children they want.^1, 2^ Contraceptive practices entail contraceptive use or non-use, discontinuation of contraception and/or failure to use any of the contraceptive methods according to a specified set of guidelines.^3^

In the developing world, almost 137 million women who want to avoid pregnancy do not use any method of family planning.^4, 5^ Of the 210 million pregnancies that occur each year, almost 80 million are unplanned. Each year, approximately 42 million pregnant women seek termination of their unplanned pregnancy. Of the 20 million women who undergo unsafe abortions by untrained staff, 67 000 die annually. Almost all of these deaths (99%) occur in underdeveloped countries.^6^

Two nationally-representative surveys in Britain and Germany revealed that 67% of British and 96% of German women used contraception,^4^ although contraceptive use patterns differed between East Germany, West Germany and Great Britain. Oral contraceptive use declined with age in both countries. Intrauterine device use and sterilisation increased with age. In Britain, oral contraceptive use was greater in urban areas than in rural areas. Intrauterine contraceptive device use and sterilisation rates were higher and oral contraceptive use rates were lower amongst married women as compared with never-married women. Increased church attendance was associated with a higher rate of condom usage. In Europe, there was no clear association between employment status and contraceptive practices.^4^

In South Africa, where contraceptives are provided free of charge, 30% to 50% of women present with unwanted and unplanned pregnancies and then seek termination of pregnancy.^3^ In a 2009 report on Nigerian women, 31.5% believed that having sex once with a man would not result in pregnancy, although 90.0% knew about the benefits of family planning. Consideration of personal health and husband's approval were major determinants regarding the respondents’ use of contraceptives. The authors concluded that there was a need for continuous education about contraception.^7^

From the literature, it is seen that contraceptive practices vary in Europe, South Africa and Nigeria and are influenced by the region in which the women live. In addition, whether they live in developing or developed countries also influences their practices, although these vary even in countries with similar economic statuses and amongst women of different age groups, education, social factors, marital status and religion.^4, 5, 7^

Sub-Saharan Africa has the highest fertility rate in the world, namely, 5.5 births per woman. Singh reports that one in every three births is involuntary.^5^ The 1998 South African Demographics and Health Survey (SADHS) reported that 96% of women knew about contraception, although the rate of use of contraception was approximately 50%.^8^ The conclusion of the study was that the low rate of condom use could be a result of lack of knowledge regarding how to use a condom, where to get condoms and the benefits of condom use. The SADHS study reported a decline in the level of knowledge of contraceptive methods from 1998 to 2008. When looking at married women, more than half (60%) used some method of contraception, with modern methods of contraception being used almost exclusively. Less than 0.1% of women relied on traditional methods and, of the modern methods, injectable contraception was the most widely used. Contraceptive use rose markedly as the education of the woman increased, as well as when the woman had at least three children.^9^

The traditional method of abstention is practised commonly in the Democratic Republic of Congo (DRC), resulting in a high rate of polygamy.^5^ In the DRC, the contraception rate is estimated to be 8% for all methods and 2% for modern methods.^10^ No study investigating factors influencing contraceptive use in the Vanga district was found.

Vanga health zone is one of the 515 health zones in the DRC, with the total population in the area being estimated at 244 839. In Vanga health zone, a programme of family planning was introduced in 1970 by Santé Rurale (SANRU), a USAID-funded programme for sustainable primary healthcare. However, contraception is not always available and women do not make regular use of these services. In Vanga hospital (the referral hospital), it is common to encounter diseases related to unsafe abortions, ranging from anaemia to sepsis and death. We sought to investigate the factors that influence contraceptive use amongst women of childbearing age in the Vanga health zone.

## Research methods and design

### Study design

This was a cross-sectional survey using interviewer-administered questionnaires. The questionnaire was administered by 38 trained interviewers. The questionnaire was translated from English to Kituba, then independently back translated to English, to check for distortions in the meaning and correctness of the translations.

### Study population and sampling strategy

The total number of women of childbearing age in the region (18 to 49 years) was estimated to be 51 416, as determined by census.

Using Epi-Info (version 6.3), a confidence interval of 95%, a standard error of 0.01 and power of 80%, the sample size was calculated to be 384. Pregnant women were excluded from the study.

Systematic random sampling was used in order to recruit participants.

### Data collection

Three variables were studied. These included socio-demographic variables, contraceptive practices of the women and factors leading to non-use of contraception. In assessing the practice of contraception, questions were asked about previous contraceptive use; age at first use of contraception; methods of contraception; and duration of current contraceptive use. To determine the factors influencing non-use of contraception, options presented included fear; inefficiency; partner's influence; unavailability of contraception; contraindications to usage; or non-awareness regarding contraception. The study was conducted over four months during 2009 and all women who were approached consented to participate in the study.

### Data analysis

All data were entered onto an Excel spreadsheet and another person verified the data entries. Frequencies were calculated for each variable of interest and inferential statistics was used to determine the relationship between the variables by using SPSS software version 17.0. The statistical significance level was set at *p* < 0.05

### Ethical considerations

Ethical approval for this study was obtained from the University of Limpopo Medunsa Campus Ethics Committee, MREC number MREC/M/111/2009:PG. Written informed consent was obtained from all the participants. Anonymity and confidentiality were assured. Permission was obtained from the manager of Vanga Health Zone central office.

## Results

### Baseline characteristics of the participants

The largest group amongst the 384 women recruited into the study fell into the31–40 year age group (*n* = 177; 46.1%). Two hundred and one (52%) had reached a primary school level of education. Three hundred and thirty-nine participants (88%) did not have any formal employment.

Three hundred and thirty-three of the women (86.9%) were married, 287 (74.7%) had experienced more than three pregnancies and 277 (72%) had more than three living children. The common religions were Protestant (*n* = 154; 51.6%) and Catholic (*n* = 198; 40.1%), as is shown in Table 1.

**TABLE 1:** Baseline characteristics of the participants.

Baseline characteristics	Number	Percentage
**Age group in years**		
18–20	12	3.1
21–30	145	37.7
31–40	177	46.1
41–49	50	13.1
**Occupation/Training**		
Tailor	17	4.4
Nurse	60	15.6
Saleslady	26	6.7
Teacher	281	73.3
**Educational level**		
Primary school	201	52.3
High school	161	41.9
University	18	4.7
No education	4	1.0
**Religion**		
Catholic	154	40.1
Protestant	198	51.6
Muslim	2	0.5
Other religion	30	7.8
**Marital status**		
Married	333	86.9
Unmarried	51	13.1
**Number of previous pregnancies**		
0	12	3.1
1–2	95	22.1
≥ 3	287	74.7
**Number of live children**		
0	12	3.1
1–2	85	24.7
≥ 3	277	72.1
**Tribe**		
Mbala	186	48.4
Yansi	84	21.9
Muhungana	38	9.9
Songo	61	15.9
Other	15	3.9
**Currently employed in the formal sector**		
Yes	45	12

One hundred and forty participants (36.5%) reported the current use of contraception, with 60% (*n* = 230) of those using modern methods of contraception; 61 (36.1%) of them had begun using contraception before the age of 20 (Table 2); and the most common methods of contraception were the oral contraceptive pill and injection, both 22.9% (*n* = 32).

**TABLE 2:** Contraception use amongst women of reproductive age.

Descriptor	Number	Percentage
**Age at the first use of contraception**		
18–20	61	36.1
21–30	46	27.2
31–40	47	27.8
41–49	15	8.9
**Contraceptive methods currently used**		
Combined contraceptive oral pills	10	7.1
Male condom	32	22.9
Female condom	3	2.1
Tubal ligation	7	5.0
*Coitus interruptus*	35	25.0
Calendar method	20	14.3
Injectable hormonal method	32	22.9
**Number of women using contraception according to age (** ***n*** **= 157)**		
18–20	50	31.9
21–30	30	19.1
31–40	41	26.1
41–49	36	22.9
**Women using contraception according to educational level (** ***n*** **= 140)**		
Primary school	71	50.7
High school	63	45
University	4	2.9
No formal education	2	1.4

### Characteristics of women taking contraception

One third of the current users of contraception (*n* = 31.9%) were younger than 20 and the largest group (*n* = 30; 21.4%) had been using contraception for anything from 13 months to two years. There was a wide variation in the duration of contraceptive use. The main reason for using contraception was for the spacing of children.

Seventy-seven (31.5%) of the women reported that they did not use contraception because of a fear of side effects (Table 3).

**TABLE 3:** Characteristics of women taking contraception.

Variable	Number	Percentage
**Proportion of women using contraception according to marital status**		
Married (*n*= 333)	123	37
Single (*n* = 51)	16	32
**Use of contraception and number of children(** ***n*** **= 140)**		
0	4	2.9
1–2	27	19.3
≥ 3	109	77.8
**Contraception use and Tribe** ***(n*** **= 133)**		
Mbala	69	52
Yansi	26	19.5
Muhungana	12	9
Songo	26	19.5
**Duration of contraception use (** ***n*** **= 140)**		
≤ 3 months	18	12.9
4–6 months	14	10
7–9 months	20	14.3
10–12 months	15	10.7
13 months–2 yrs	30	21.4
25 months–4 yrs	20	14.3
≥ 4 yrs	23	16.4
**Reason for contraceptive use**		
Does not want more children	38	27.1
Normal vaginal delivery is contraindicated	7	5
Pregnancy makes the woman very sick	15	10.7
Family spacing	63	45
Other reason	17	12.1
**Reasons for not using contraception**		
Fear of side effects	77	31.5
Fear of infertility	26	10.6
Distrust contraception	8	3.3
Husband's disapproval	38	15.6
Unavailability of the desired contraception	12	4.9
Complete absence of contraceptive methods.	17	7.0
Has contraindications to contraceptive use	4	1.6
Too expensive	44	18.0
Her religion disapproves use	18	7.4

Injectable contraception was used only by women who had children (Table 4).

**TABLE 4:** Types and contraception used by women.

Variable	Oralcontraception (%)	Malecondom (%)	Femalecondom (%)	Tuballigation (%)	*Coitusinterruptus* (%)	Calendarmethod (%)	Injectablemethods (%)
**Tribe & type of contraception used**							
Mbala	4	22	1	3	17	10	13
Yansi	3	3	1	0	10	4	8
Muhungana	1	1	0	3	4	3	3
Songo	3	7	0	1	5	3	8
**Number of live children & type of contraception used**							
0	0	1	0	0	2	1	0
1 or 2	1	10	0	0	8	7	2
≥ 3	9	21	2	7	25	13	30
**Religion**							
Protestant	5	20	3	8	26	14	21
Catholic	11	24	0	2	28	15	23

### Correlation analysis

Statistical analysis showed strong statistically-significant relationships between past use of contraception with age (*p* = 0.000) and number of children (*p* = 0.000). The comparison between non-use of contraceptives with age (*p* = 0.005) and the number of children (*p* = 0.002) were also of statistical significance (Table 5).

**TABLE 5:** Correlations analysis.

Variable	Pearson's correlation coefficient	*p*-value
**Past use of contraception**		
Age	-0.203	0.000*
Number of children	-0.213	0.000*
Number of live children	-0.173	0.001*
**No practice of contraception**		
Age	0.143	0.005*
Number of children	0.161	0.002*
Number of live children	0.139	0.006*

* , Statistically significant.

## Discussion

This study sought to understand contraceptive use amongst women of childbearing age in the Vanga province of the DRC. This is very relevant in the DRC as well as Africa, in general, given the HIV epidemic and the high rate of unplanned pregnancy.

Many of the participants were in the 31 to 40 year age group (46.1%). Women aged 15 to 20 were more likely to use contraception (50%).

A demographic and health survey conducted in the DRC during 2007 showed that amongst sexually-active women, contraception use was as follows: 20% for women younger than 20, 39% for those aged 20–29, 24% for those aged 30–39 and 17% for those aged 40–49.^11^ This differed from our findings since younger women in the Vanga region are now more aware of HIV and the benefits of contraception than five years ago, when the survey was conducted.

Slightly more than half (52.3%) of the 384 participants had primary school education, whereas four of the participants had no formal education. Of the women who had only a primary school education, 35% were using contraception, whilst only 22% of the women with University level education used contraception. In our survey, we had very few respondents who had University level education, however the proportion of these participants using contraception is low. It would thus seem that higher education is not a predictor for contraceptive use amongst these participants.

Most of our participants (88%) were without formal employment. This is consistent with most rural settings in sub-Saharan Africa. During the demographic and health survey in the DRC in 2007, it was noted that the percentage of women who worked increased with age, where 81% of the 40–49 year age group were employed, whilst only 35% of those in the 15–19 year age group were employed.^11^ The survey in 2007 found that 25% of all employment was in the sales and services sector,^11^ which is consistent with our findings.

Christians made up the majority (92%) of the participants, with the most common denominations being Protestant (51.6%) and Catholic (40.1%). Our findings were consistent with results from the national survey, where 93% of women surveyed were Christian.^11^ Natural contraceptive methods such as abstinence and the rhythm method are permissible for Catholics. Amongst Protestants, it is common for adherents to use birth control after the family is complete. Family planning was used for birth spacing rather than to restrict the overall size of families.^7^

In our study, 86% of the women were married. In the DRC as a whole, about 58% of all women are married.^11^ This difference may be because early age marriages are common in Vanga. We found that contraceptive use was higher amongst married participants, whereas according to the national survey, contraception was used more by single women (69%) and male condoms were used most often.^11^

There was no significant difference between married and single participants regarding preference in terms of contraceptive methods. Dibaba reported that only 13% of married women in Asia used contraception.^12^

Of the women surveyed, 74.5% had experienced more than three child births and, of these, 72% had more than three children who were still alive. Tehrani, Farahani and Hashemi found that 50% of the women had one living child, 36.4% had two children and 11.7% had three. In the DRC, however, large families are common.^13^

Most participants were from the Mbala tribe (48.4%). This is the largest tribe in this region and members of the tribe use all forms of contraception, indicating that belonging to this tribe does not seem to prevent women from using any particular form of contraception.

One hundred and sixty-eight (43.7%) of the participants reported having used contraception in the past and 36.3% of them had started using contraception before they were 20 years old. The national study found that about half of the participants (49%) had used at least one contraceptive method at some time in their lives.^11^ In the study conducted by Bhatti in1995,^14^ 5% had initiated contraceptive use at ages 15–19 years; 22% at ages 20–24 years; and 58% at ages 25–34 years. Our findings are thus similar to the national study in the DRC, in that slightly less than half of the women surveyed had ever used contraception.

In our survey, 36.4% of the women reported they were currently using contraception. Other research found that contraceptive use is similar in Kinshasa (42%) and Bas-Congo (40%).^11^

Sixty percent of the women in our study used modern contraceptive methods such as male condoms and injectable contraceptives (22.9% each). Tehrani, Farahani and Hashemi reported that natural methods (withdrawal and rhythm) were the most commonly-used contraceptive methods (61.0%), whilst the use of oral contraceptives, intra-uterine devices and condoms were at 22.9%, 10.1% and 6.0%, respectively.^11^ From the demographic and health survey, the male condom (15%), oral contraceptive pill (4%) and injection (1%) were the most commonly-used modern contraceptive methods. Periodic abstinence (38%) was the most frequently-used traditional method, followed by withdrawal (23%).^11^ It would thus seem that more women in the Vanga district use injectable hormones and male condoms as contraceptives as compared with the rest of the DRC. This may relate to the fact that SANRU has made these methods available to women in the Vanga district. A study by Masoda and Govender found that contraception price was a factor restricting its use in the DRC.^15^

We found that injectable contraceptive methods were the first choice amongst the women who had more than three children, followed by the male condom; whilst male condoms were the most common amongst women with one to two children and interrupted coitus was most common in the group without any children. The male condom was the most commonly-used method amongst the group of Mbala participants, whilst interrupted coitus was the most common method amongst Yansi and Muhungana participants. In the group of Songo participants, the most common method used was injectable contraception.

The intra-uterine device was the preferred method amongst women of childbearing age in Pakistan, followed by injectable contraceptives, oral contraceptive pills and male condoms. Only a minority (4%) of women preferred the use of the non-medical (traditional) methods of calendar and *coitus interuptus*.^14^ In the United States, the most common methods used were the male condom (used at least once by 94% of study participants) and the pill (used at least once by 61%).^16^ According to Berer, condoms are the mainstay of dual protection and will remain so until such time as other equally safe and effective methods are developed.^17^

Most participants (21.4%) have been using contraceptives for a short period of time (one to two years). This may be because of the increased involvement of non-governmental organisations, (NGOs) such as Santé Rurale (SANRU), over the past three years. Most of these NGOs are religion based and their activity has increased as the war in the DRC is stabilising. Many of the participants (45%) used contraception for spacing of their children.

The main reasons for not using contraceptives were a fear of side effects (31%), followed by disapproval of the husband (15%). The demographic and health survey found that women do not use contraceptives because of side effects (6%), medical contraindications (3%), refusal of the husband/partner (5%) and religious restrictions (6%).^11^ In our study, 7% said they did not use contraception because it was not allowed by their religion. Roman Catholics are forbidden to use medical or physical contraceptive methods.

### Implications and recommendations

Women have been the primary subject of contraceptive and family planning research because most modern contraceptive methods were designed for use by women and because most programmes assume that women are primarily responsible for family planning.^18^ However, a large proportion of women (15.6%) do not use contraception in the Vanga district because it is not permitted by their male partners, husbands in most instances. Cost of contraception is also a barrier to many women using contraception as it is free only at the non-governmental clinic (SANRU) and is sometimes out of stock, even at these clinics. This has also been found as a barrier in another study in the DRC.^15^

The participants’ age and number of children impacts on their contraceptive use. The more children and the older a woman is, the more likely she is to use contraception. One of the contributing factors for this could be exposure to health education at ante-natal clinics and facilities located at the community level. The demographic and health survey also found that women with more children were more likely to use contraception.^8^ The use of contraception was very low amongst woman with no children.^11^ Tehrani, Farahani and Hashemi found in Tehran that age, women's level of education, their husbands’ level of education and previous familiarity with contraceptive methods were the most important factors influencing contraceptive use.^11^ A review conducted by Bongaarts and Bruce showed that socio-economic, behavioural and cultural factors influenced contraceptive use.^19^

Increasing the education of women regarding contraception and the various methods available could help to increase the use of family planning as we have found education to be an important predictor of contraception use. The other barriers to the use of contraception were religion and the objection of a woman's partner to contraceptive use. Male partners need to be involved actively in the choice and use of contraception and should be present during the consultation. It would be informative to carry out a similar study amongst the men in order to discover their views regarding contraceptive use. In this way, education which is designed to empower women may have a twofold effect. Firstly, women will become more aware of contraception and the choices available and, secondly, women will be empowered economically so they can be seen as equals in the Vanga district.

### Limitations

Systematic sampling may have reduced the validity of the results. Using interviewers to assist in the completion of the questionnaires may have distorted the validity of responses as some participants may not have felt comfortable, thus giving responses that they thought were acceptable. We tried to reduce this by training the interviewers and asking them to maintain self-awareness and to demonstrate ‘neutrality’ during the interviews.

## Conclusion

The main reason for women in this district to use contraception was for family spacing and women who had used contraception in the past were more likely to use it again. The fear of side effects was a major barrier to contraceptive use. Contraception methods and education regarding their use should be made easily accessible throughout the Vanga Health Zone.
